# Identification of lethal species in *amanita* section *Phalloideae* based on nucleotide signature and specific TaqMan-MGB probe and primer

**DOI:** 10.3389/fmicb.2024.1301085

**Published:** 2024-02-01

**Authors:** Renhe Duan, Jiahui Huang, Donghan Zhang, Enjing Tian

**Affiliations:** Country Engineering Research Center of Edible and Medicinal Fungi, Ministry of Education, Jilin Agricultural University, Changchun, China

**Keywords:** internally transcribed spacer, lethal poisonous mushrooms, rapid detection, real-time PCR, specific DNA sequences

## Abstract

*Amanita* section *Phalloideae* consists of lethal toxic mushroom species, causing many fatal poisoning incidents worldwide. Molecular techniques of nucleotide signatures and single nucleotide polymorphism (SNP) detection could be used to develop a specific method for identifying lethal section (sect.) *Phalloideae* species. A comparison of 38 sequenced and 228 validated sequences from sect. *Phalloideae* species showed a 17-base pair nucleotide signature and an SNP site between the lethal and non-lethal species. A specific minor groove binder probe was designed based on them. The results indicated that this method exhibited excellent specificity for the lethal subgroup, good detection in samples subjected to simulated gastric digestion (60 min boiling and 120 min digestion), and a 10 pg./μL detection limit. This method enables accurate detection of target species in samples under complex conditions and can provide evidence for poisoning incidents caused by lethal sect. *Phalloideae* species to assist in targeted treatment strategies.

## 1 Introduction

Mushrooms are widely consumed by humans due to their high palatability, nutritional value, and health-promoting abilities (Zhao et al., 2020; Venturella et al., 2021; Yadav and Negi, 2021). However, the general public lacks the ability to identify toxic mushrooms, which has led to the frequent occurrence of poisoning incidents arising from the inadvertent ingestion of toxic mushrooms (Li et al., 2023). In particular, the ingestion of toxic species within the genus *Amanita* accounts for more than 90% of deaths caused by accidental toxic mushroom ingestion (Bresinsky, 1990). In a study that investigated 102 cases of mushroom poisoning in Southern China, it was found that mushrooms of the genus *Amanita* accounted for 70.49% of the fatalities (Chen et al., 2014), with a considerable proportion attributed to species belonging to *Amanita* section (sect.) *Phalloideae* (Gausterer et al., 2014).

*Amanita* section *Phalloideae* is an important group of poisonous mushrooms in the genus *Amanita*, belonging to the family Amanitaceae in Fungi (Cui et al., 2018). Chinese researchers have reported that a total of 15 species belong to this section (Zhang et al., 2010; Cui et al., 2018), with the highly toxic members containing amatoxins, phallotoxins, and virotoxins (Fu et al., 2017). The α-amanitin and phallacidin genes believed to be responsible for the synthesis of lethal toxins are found in the toxic species of *A*. sect. *Phalloideae*. However, they are absent in the non-toxic species of the section (Hallen et al., 2007). A genome-guided approach was confirmed to be reliable for identifying novel cyclic peptides in lethal *Amanita* species (Zhou et al., 2021). The evolution of the biosynthetic pathway for amanitin from lethal *Amanita* species generates a more extensive array of harmful cyclic peptides (Luo et al., 2022). The ingestion of toxic mushrooms belonging to *A*. sect. *Phalloideae* is often accompanied by typical symptoms such as nausea, vomiting, abdominal pain, and diarrhea. Therefore, it is often mistaken for gastroenteritis-type toxic mushroom poisoning. Such misdiagnosis may lead to delayed treatment, which may cause liver and kidney failure within days after ingestion and ultimately result in death (Broussard et al., 2001). A case study deduced that oral intake of 0.2 mg/kg α-amanitin can be lethal (Yilmaz et al., 2015). The α-amanitin content in *A. subjunquillea* S. Imai belonging to sect. *Phalloideae* reached 2.3959 mg/g (Bao et al., 2005).

In China, dozens of individuals die yearly from inadvertent ingestion of toxic species of *Amanita* sect. *Phalloidea.* (Li et al., 2020, 2022a, 2023; Li H. et al., 2021; Li W. et al., 2021). Among the 203 mushroom poisoning incidents that occurred in Changsha City, China, from 2016 to 2020, there were a total of 15 fatalities, with the most deaths attributed to *A. fuliginea* Hongo and *A. rimosa* P. Zhang and Zhu L. Yang, both members of *A.* sect. *Phalloideae* (Ma et al., 2022). Investigation of 223 poisoning incidents that occurred in Yunnan Province since 2013 found that the lethal species *A. exitialis* was responsible for 19 poisoning incidents in five cities/prefectures, resulting in it being the deadliest mushroom in Yunnan Province (Li et al., 2022b). An analysis of the poisoning incidents caused by the common poisonous *Amanita* species in Guangdong Province between 2000 and 2019 revealed that *A. exitialis* Zhu L. Yang and T.H. Li caused the most fatal poisoning incidents, being responsible for the death of 44 individuals (73.33% of all fatalities), followed by *A. rimosa*, *A. fuligineoides* P. Zhang and Zhu L. Yang, and *A. fuliginea*. All deaths in this analysis were caused by the *A*. sect. *Phalloideae* species (Deng et al., 2020).

Inadvertent ingestion of toxic mushrooms is mainly caused by a preference for wild foods coupled with a lack of the ability to distinguish between toxic and non-toxic species; it is difficult for nonprofessionals without systematic training to identify toxic and non-toxic mushrooms. In addition, undocumented toxic species are often encountered during wild mushroom gatherings (Broussard et al., 2001). In the unfortunate event of mushroom poisoning, accurate diagnosis, prompt intervention, and treatment are very important. Generally, the most effective method for handling poisoning incidents involves the initial identification of specimens to confirm the ingested species (Yilmaz et al., 2015). Therefore, there is a need to develop an accurate and rapid method for identifying lethal species of *Amanita* sect. *Phalloideae* to assist healthcare institutions and disease control centers in the swift and effective diagnosis of mushroom poisoning and formulating subsequent treatment regimens, which will significantly enhance treatment effects.

The concept of deoxyribonucleic acid (DNA) barcoding, inspired by product barcodes, was first advocated by Arnot et al. (1993). The aim is to use a standardized short gene sequence (the DNA barcode) to rapidly identify species (Hebert et al., 2003). The emergence of the universal Internally Transcribed Spacer (ITS)1 and ITS4 primers in fungal research indicates that ITS sequences can serve as highly versatile DNA barcodes (White et al., 1990). However, this does not mean that ITS sequences can be applied to all fungal identification processes. Low-quality DNA templates are commonly encountered in practical applications, which poses difficulties for the successful amplification of target fragments (Bauer et al., 2003). Mini-barcode technique as a supplement to DNA barcoding can overcome identification problems caused by DNA degradation. The study’s results indicated that amplification success rates of more than 90–95% were obtained from animal samples with DNA degradation (Meusnier et al., 2008).

A study by Jianping Han’s research team in 2016 first proposed the concept of nucleotide signatures for traditional Chinese medicine (TCM) materials based on the mini-barcode technique. The nucleotide signatures are extremely short (20–50 bp) species-specific DNA sequences and have been applied to identify various TCM materials (Liu et al., 2016). A nucleotide signature developed for Wuweizi (*Schisandrae chinensis* Fructus) was demonstrated to be served as an effective tool for identifying Wuweizi and its derived Chinese patent medicines (CPMs) (Jiang et al., 2019). Maidong (Ophiopogonis Radix, from the dried root tuber of *Ophiopogon japonicus*) and its derived CPMs were successfully authenticated using the nucleotide signature approach (Guo et al., 2020). Furthermore, a nucleotide signature for the genus *Aconitum*, a genus-level nucleotide signature, was developed to be used to identify the *Aconitum* species, which enabled a considerable expansion of the application of nucleotide signatures (Wang et al., 2022b). The identification of ephedra-containing products was also implemented using a genus-level nucleotide signature (Wang et al., 2022a), which indicates that this technique has been extensively applied in the identification of TCM materials and possesses a wide range of applications. Recently, the techniques of the species-level nucleotide signatures were also applied to the identification of poisonous mushrooms (Gao, 2022; Xie, 2022; Xie et al., 2022).

**Figure 1:**
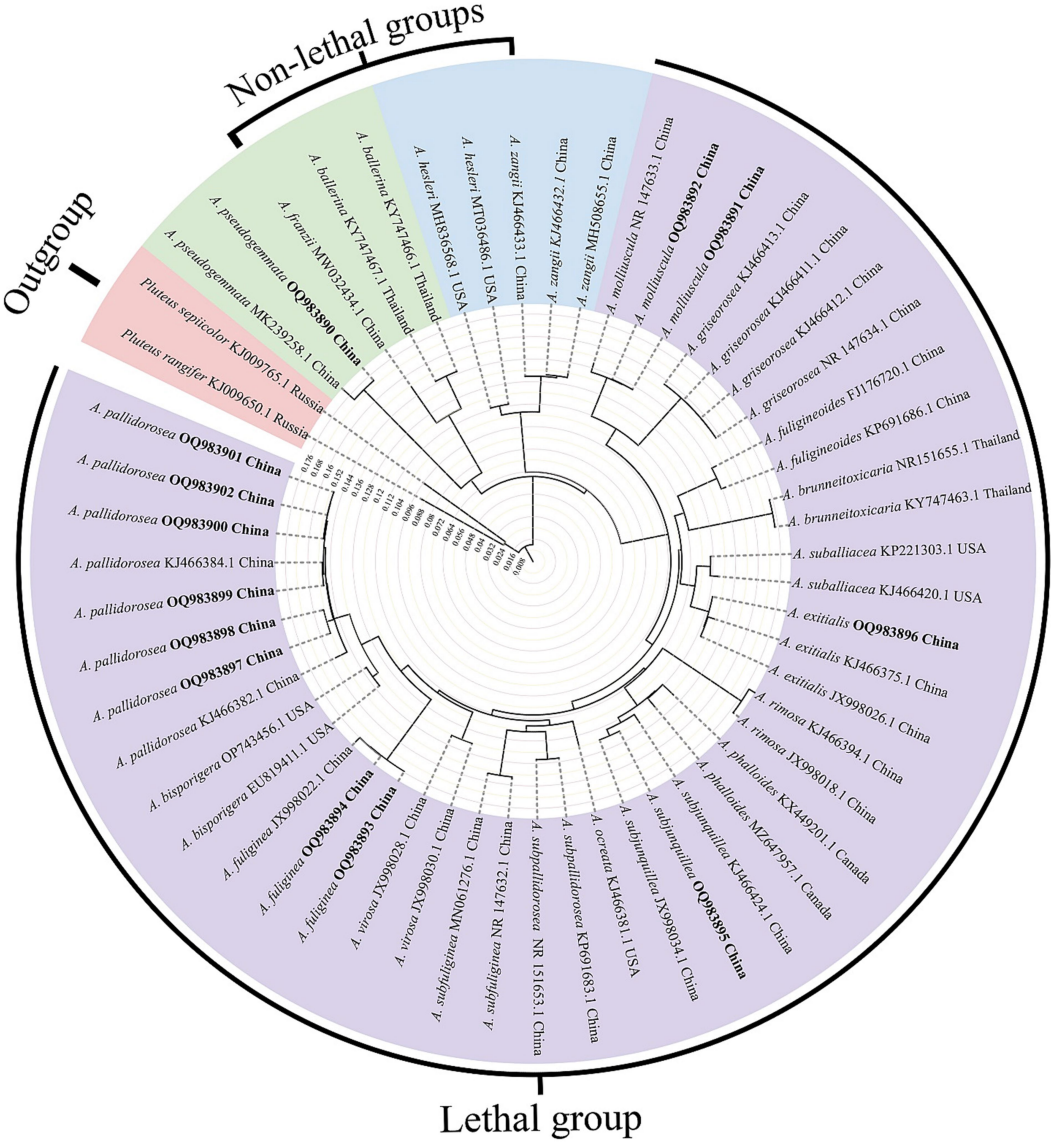
Neighbor-Joining (NJ) phylogram of *Amanita* sect. *Phalloideae* inferred from the ITS dataset. Self-test sequences are in bold. The red block is the outgroup, the green and blue blocks are two non-lethal subgroups, and the purple block is the lethal subgroup.

In this study, a specific nucleotide signature for *Amanita* sect. *Phalloideae* was developed. Furthermore, in our systematic diversity study of *A.* sect. *Phalloideae*, lethal, and non-lethal *Amanita* species gathered in distinct subclades as shown in Figure 1. Through the extensive sequence analysis, a single nucleotide polymorphism (SNP) site located in the 5.8S region was found between the two subgroups. Hence, based on the section-level nucleotide signature and the SNP site, an accurate, efficient, rapid and sensitive method was established for the detection of the lethal *Amanita* sect. *Phalloideae* species, which is of great significance for the diagnosis and targeted treatment of poisoning cases caused by these toxic mushrooms.

## 2 Materials and methods

### 2.1 Materials

**Figure 2:**
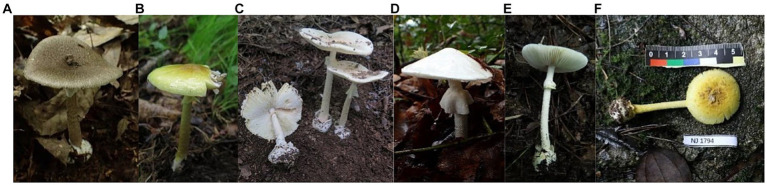
Photographs of *Amanita* sect. *Phalloideae* species collected in this study. **(A)** *Amanita fuliginea* Hongo; **(B)** *Amanita subjunquillea* S. Imai; **(C)** *Amanita pallidorosea* P. Zhang and Zhu L. Yang; **(D)** *Amanita exitialis* Zhu L. Yang and T.H. Li; **(E)** *Amanita molliuscula* Q. Cai, Zhu L. Yang, and Y.Y. Cui; **(F)** *Amanita pseudogemmata* Hongo. Photos by Enjing Tian and Junqing Yan.

The materials used in this study were collected in the field or borrowed from herbaria (Table 1). A total of 23 mushroom species were used, including 16 species of the genus *Amanita* and seven species of other genera. These *Amanita* species included seven *Amanita* sect. *Phalloideae* species, with five being lethal species. All samples were systematically verified morphologically and molecularly and preserved at the Herbarium of Mycology of Jilin Agricultural University (HMJAU) and Herbarium of Cryptogams Kunming Institute of Botany Academia Sinica (HKAS). The photos of partial *Amanita* sect. *Phalloideae* species in this study were illustrated in Figure 2.

**Table 1:** Information of materials used in this study.

No.	Species	Voucher	Collection site	*Amanita* sect. *Phalloideae* species	Lethal *Amanita.* sect. *Phalloideae* species
1	*Amanita fuliginea*	HMJAU64348	Jiangsu, China	Yes	Yes
2	*Amanita subjunquillea*	HMJAU64359	Henan, China	Yes	Yes
3	*Amanita pallidorosea*	HMJAU64358	Henan, China	Yes	Yes
4	*Amanita exitialis*	HMJAU64352	Guangdong, China	Yes	Yes
5	*Amanita molliuscula*	HMJAU64597	Jilin, China	Yes	Yes
6	*Amanita pseudogemmata*	HMJAU64730	Zhejiang, China	Yes	No
7	*Amanita franzii*	HKAS91231	Yunnan, China	Yes	No
8	*Amanita farinose*	HMJAU64366	Jilin, China	No	No
9	*Amanita fritillaria*	HMJAU64654	Hunan, China	No	No
10	*Amanita ibotengutake*	HMJAU64601	Jilin, China	No	No
11	*Amanita kotohiraensis*	HMJAU64364	Jilin, China	No	No
12	*Amanita longistriata*	HMJAU64569	Jilin, China	No	No
13	*Amanita orientifulva*	HMJAU64687	Guangxi, China	No	No
14	*Amanita orientigemmata*	HMJAU64691	Guangxi, China	No	No
15	*Amanita manginiana*	HMJAU64518	Henan, China	No	No
16	*Amanita melleiceps*	HMJAU44567	Henan, China	No	No
17	*Lepiota brunneoincarnata*	HMJAU64715	Jiangsu, China	No	No
18	*Russula subnigricans*	HMJAU64266	Zhejiang, China	No	No
19	*Chlorophyllum molybdites*	HMJAU64714	Jiangsu, China	No	No
20	*Entoloma omiense*	HMJAU64346	Jiangsu, China	No	No
21	*Gymnopilus dilepis*	HMJAU47399	Chongqing, China	No	No
22	*Hypholoma fasciculare*	HMJAU64659	Hunan, China	No	No
23	*Russula japonica*	HMJAU28627	Zhejiang, China	No	No
NTC	ddH_2_O	NTC	-	No	No

NTC, no-template control, dd, deionized distilled.

The ITS sequences used for included 38 sequenced sequences from the samples in this study and 228 downloaded ones from the National Center for Biotechnology Information (NCBI) database. The downloaded sequences used for the sequence analysis were from authoritative taxonomic studies (Cui et al., 2018). The information of the downloaded and sequenced sequences is shown in Supplementary Table S1.

### 2.2 Genomic DNA extraction

Specimens that were accurately identified by morphology were subjected to total genomic DNA extraction using a modified alkaline lysis method. The alkaline lysis buffer was prepared with sodium hydroxide, 1% polyvinylpyrrolidone, and 1% Triton X-100. A total of 1–5 mg of each mushroom sample (typically mushroom lamellae) was used for DNA extraction. The alkaline lysis buffer (20 μL) was added to the sample, and the mixture was vortexed for 10–15 s. The mixture was then heated at 60°C for 2–3 min. After the addition of 180 μL 1× tris (hydroxymethyl) aminomethane (Tris) − ethylenediaminetetraacetic acid (EDTA) (TE) buffer pH 8.0, deoxyribonuclease (DNase) and ribonuclease (RNase) free, Sangon Biotech Co., Ltd., Shanghai, China, the mixture was gently vortexed to achieve uniform mixing, and the supernatant was collected for PCR. A modified cetyltrimethylammonium bromide method was employed for specimens that showed poor results with the extraction kit (Doyle and Doyle, 1987). DNA concentration and purity were measured using a NanoDrop 2000 uLtra-micro spectrophotometer (Thermo Fisher Scientific Inc., Waltham, MA, United States). After measurement, the extracted DNA was diluted to a concentration of 10 ng/μL and stored in a freezer at −20°C.

### 2.3 Primer design, PCR amplification, and sequencing

Polymerase chain reaction (PCR) was performed in a 25 μL system containing 12.5 μL of 2 × PCR Master Mix (Sangon Biotech Co., Ltd., Shanghai, China), 1.0 μL of forward primers (ITS1: 5′–CTTGGTCATTTAGAGGAAGTAA–3′)/reverse primers (ITS4: 5′–TCCTCCGCTTATTGATATGC–3′) (2.5 μM, Sangon Biotech Co., Ltd., Shanghai, China), and 1.0 μL of DNA templates; the volume was then made up to 25 μL with double-distilled water. PCR conditions were as follows: 94°C for 3 min; followed by 33 cycles of denaturation at 94°C for 30 s, annealing at 54°C for 40 s and elongation at 72°C for 40 s; and a final extension at 72°C for 10 min. The PCR products were examined via 1% agarose gel electro phoresis and sent to a sequencing company (Sangon Biotech Co., Ltd., Shanghai, China) for bi-directional sequencing based on the Sanger sequencing method.

**Figure 3:**
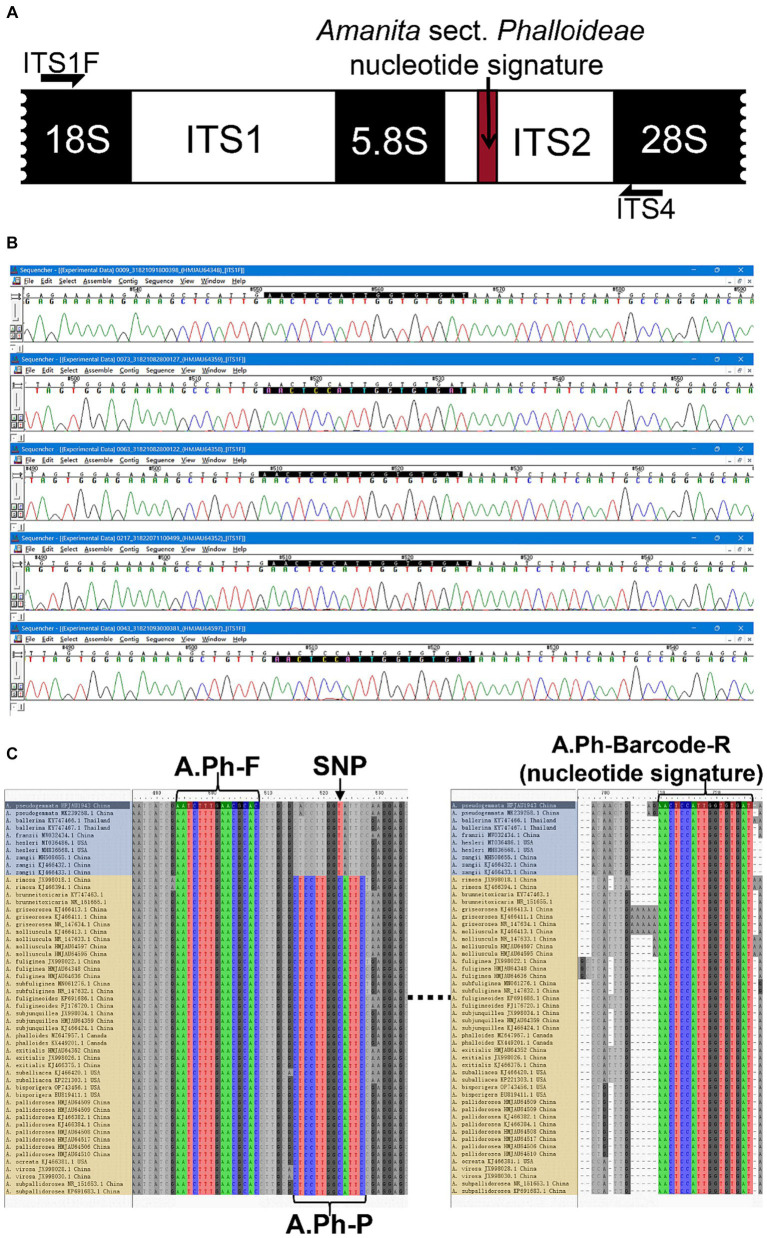
The development of a nucleotide signature for *A*. sect. *Phalloideae*. **(A)** Nucleotide signature position in the sequence. **(B)** Sequencing results of five batches of *A*. sect. *Phalloideae*. **(C)** Positions of the SNP site, primers, and probes in the sequence. The non-lethal *A.* sect. *Phalloideae* subgroup is marked in blue and the lethal *A.* sect. *Phalloideae* subgroup is marked in yellow.

Based on the ITS sequences of species within *Amanita* sect. *Phalloideae* sequenced in this study and downloaded from the NCBI database, the nucleotide signature of *A*. sect. *Phalloideae* found through specific validation was used as the downstream primer (A.Ph-Barcode-R: 5′–ATCACACCAATGGAGTT–3′). The differential SNP site between lethal and non-lethal *A*. sect. *Phalloideae* species served as the center position of the probe, and a 13 bp shared sequence of lethal *Amanita* species was screened to form the probe (A.Ph-P: 5′–FAM-CTCCTTGGCATTC-MGB–3′). Given that 13 bp is close to the lower limit of the recommended probe length, the 3′ end of the TaqMan probe was modified with an minor groove binder (MGB) group to increase the probe melting temperature (Tm). This allows TaqMan MGB probes to be considerably shorter than traditional probes, which provides better sequence discrimination ability and flexibility and allows the application to a wider range of targets. Following probe design recommendations, Primer Premier 5 (PREMIER Biosoft, Palo Alto, CA, United States) was used to design a universal upstream primer (A.Ph-F: 5′–AATCTTTGAACGCAC–3′) for *A*. sect. *Phalloideae* in the 5.8S region based on the determined downstream primer and probe. This primer design method, using a nucleotide signature as the primer at one end, ensured that the subsequent amplification reaction was specific to *A*. sect. *Phalloideae.* The assurance of intra-group specificity greatly reduced the difficulty in designing specific probes for the lethal *Amanita* subgroup within the section and dual-specific amplification enabled the improvement of detection accuracy. The positions of SNP sit, primers, and probes are shown in Figure 3C. Real-time PCR was performed in a 10 μL system. The concentrations and volumes of the reagents used in the real-time PCR reaction system are shown in Table 2.

**Table 2:** Concentration and volume of reagents used in the Real-time PCR reaction system.

Component	Concentration (μmol/L)	Volume (μL)	Final concentration (μmol/L)
qPCR Probe Mix (high ROX)	–	10	–
A.Ph F	10	1	0.5
A.Ph-Barcode R	10	1	0.5
A.Ph P	10	0.5	0.25
Template DNA	–	1	–
ddH_2_O	–	6.5	–

### 2.4 Validation of specificity

As shown in Table 1, the specificity of real-time PCR for the lethal subgroup within *Amanita* sect. *Phalloideae* was tested on the following materials: five lethal species within *A*. sect. *Phalloideae* (No. 1–5), two non-lethal species within *A*. sect. *Phalloideae* (No. 6–7), nine species of the genus *Amanita* not belonging to *A*. sect. *Phalloideae* (No. 8–16), seven toxic mushroom species of other genera (No. 17–23), and no-template control (NTC) with deionized distilled H_2_O (ddH_2_O). The reaction conditions were: 95°C for 180 s, 40 cycles of 95°C for 10 s, 54°C for 15 s, and 72°C for 20 s (with fluorescence signal acquisition). The amplification curve for the reaction was established using the cycle number as the *x*-axis and the increase in fluorescence signal (△Rn) as the *y*-axis. A distinct amplification curve indicated a positive result (the target species has been detected), whereas the absence of an amplification curve indicated a negative result (the amplified target species was absent).

### 2.5 Simulated gastric fluid digestion

Gastric fluid digestion was simulated to mimic the process of extracting test samples from the vomit of a patient during a poisoning incident (Table 3A). *Amanita pallidorosea* P. Zhang and Zhu L. Yang (species No. 3 in Table 1) was used as the sample DNA for testing. The simulated gastric fluid (SGF) was prepared by dissolving 2.0 g sodium chloride and 3.0 g pepsin in 7.0 mL hydrochloric acid, and then the volume was adjusted to 1,000 mL with water. To simulate digestion, 30 mg of the sample was boiled in water for 10, 30, or 60 min. The boiled samples were then placed in the SGF at 37°C and incubated for 30, 60, or 120 min. Finally, the nine samples were subjected to DNA extraction, as described in Section 2.2.

**Table 3:** Parameters used for the applicability and sensitivity tests.

A: Simulated gastric digestion treatment			B: DNA concentration gradient treatment	
No.	Boiling (min)	SGF treatment (min)	Dilution multiplier	Final DNA concentration
1	10	30	10	100 ng/μL
2	10	60	10^1^	10 ng/μL
3	10	120	10^2^	1 ng/μL
4	30	30	10^3^	100 pg./μL
5	30	60	10^4^	10 pg./μL
6	30	120	10^5^	1 pg./μL
7	60	30	–	–
8	60	60	–	–
9	60	120	–	–
NTC	NTC	–	–	–

NTC, no-template control; SGF, simulated gastric fluid.

### 2.6 Validation of sensitivity

The sensitivity of the developed method was tested using *Amanita pallidorosea* (species No. 3 in Table 1). An initial DNA concentration of 100 ng/μL was subjected to 10-fold serial dilutions using DNA dilution buffer (for fluorescence PCR, Sangon Biotech). A total of six dilutions were performed (Table 3B), and the experiment was repeated three times for each dilution. A standard curve was established based on the results.

## 3 Results

### 3.1 Establishment of nucleotide signature

Extensive screening and mining revealed a lack of interspecific specificity of the ITS2 region between 5.8S and 28S rDNA in *Amanita* sect. *Phalloideae*. However, strong inter-sect. Specificity was observed, which indicated the potential for the development of a sect.-level nucleotide signature. A 23 bp conserved sequence was extracted from the ITS2 region; however, its excessive length caused high specificity, which resulted in the coverage of only a few species within *A*. sect. *Phalloideae*. Further reduction and screening of the 23 bp sequence led to the generation of a 17 bp short sequence (5′-AACTCCATTGGTGTGAT-3′) close to 28S ribosomal (r)DNA within the ITS2 region that covered all species of *A*. sect. *Phalloideae* in the extensive screening (Figure 3A). Therefore, this 17 bp short sequence possesses an immense potential to serve as a nucleotide signature sequence for *A*. sect. *Phalloideae*.

To validate the versatility of *Amanita* sect. *Phalloideae*, the 17 bp short sequence was subjected to alignment with 38 *A*. sect. *Phalloideae* sequences obtained from collected samples and 228 sequences were downloaded from GenBank (Supplementary Table S1) using the alignment viewer and editor (Aliview) software. The results indicated that all *A*. sect. *Phalloideae* sequences contained this short sequence (Figure 3C) located close to 28S rDNA within the ITS2 region in all sequences (Figure 3A). Sequencing chromatograms visualized in Sequencher 5.4.5 (Figure 3B) revealed that the peak pattern within this region was uniform and orderly. This suggested a low possibility of the presence of SNP sites within this region, which indicated high applicability and a low risk of variation.

Basic Local Alignment Search Tool (BLAST) alignment was performed with the screened nucleotide signature of *Amanita* sect. *Phalloideae* at the NCBI website. Among the 805 results with 100% similarity and coverage, except for certain non-fungal species and indeterminate fungal species, all other species belonged to *A*. sect. *Phalloideae*. This result demonstrates that the 17 bp *A*. sect. *Phalloideae* nucleotide signature provided excellent specificity in differentiating between *A*. sect. *Phalloideae* and other macrofungi. In an extensive screening effort for nucleotide signatures, we identified an SNP site in the 5.8S near the ITS2 region that can be used to distinguish the lethal from the non-lethal subgroup of *A.* sect. *Phalloideae* (Figure 3C).

### 3.2 Validation of specificity

**Figure 4:**
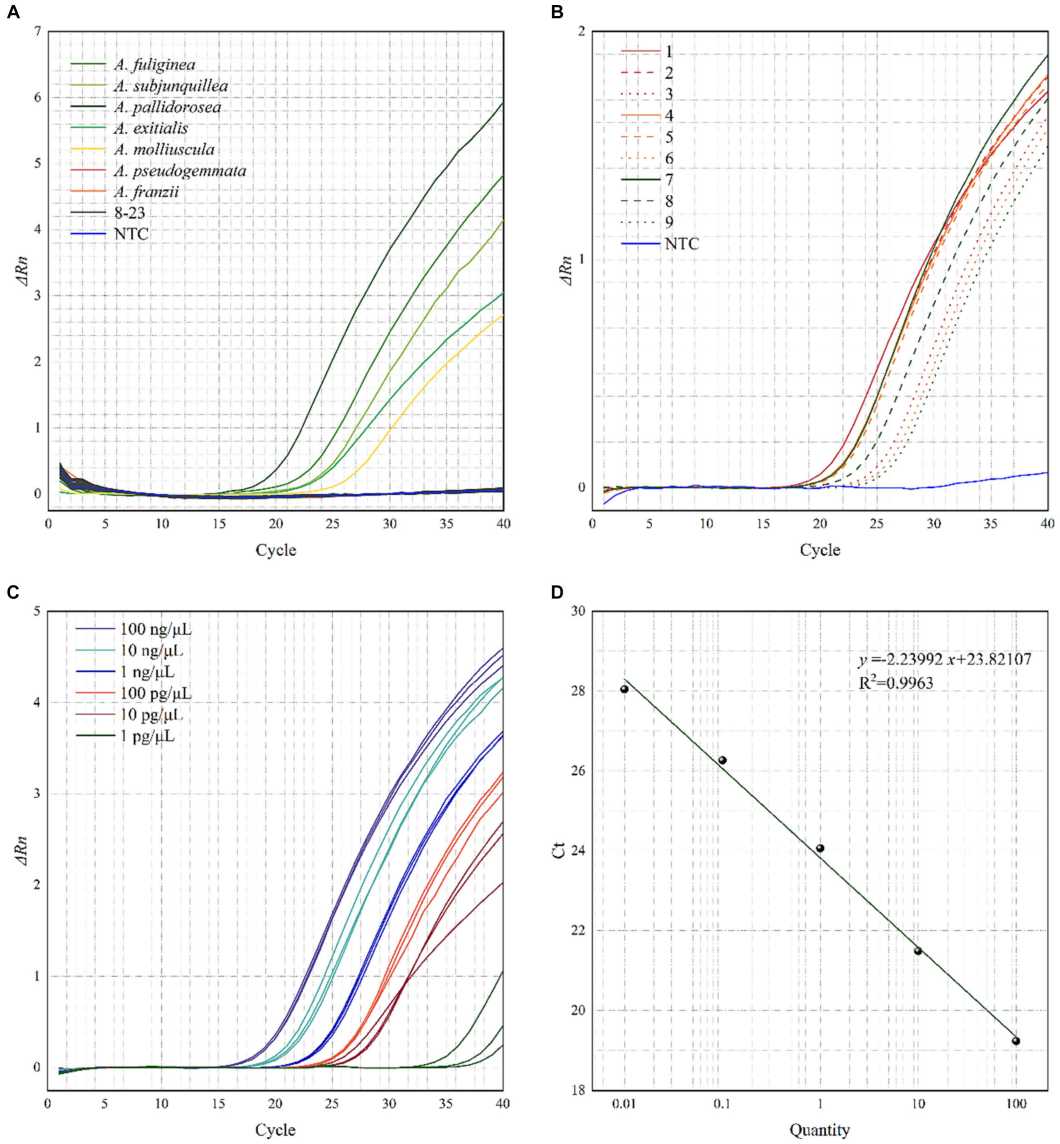
Testing the **(A)** specificity, **(B)** applicability, **(C)** relative sensitivity of the real-time PCR assays for the lethal *A*. sect. *Phalloideae* subgroup, and **(D)** Standard curve of the real-time PCR. A No. 8–16 are species of the genus *Amanita* not belonging to *A*. sect. *Phalloideae*, No. 17–23, are toxic mushroom species of other genera. B Samples with boiling treatment time of 10, 30, and 60 min are marked in red, yellow, and green, respectively, and samples with SGF treatment of 30, 60, and 120 min are marked in solid, dashed, and dotted lines, respectively.

Real-time PCR was performed on the 24 samples in Table 1, and the resultant amplification curves are shown in Figure 4A. Fluorescence signals and distinct amplification curves were present for the lethal subgroup (No. 1–5) within *Amanita* sect. *Phalloideae*, indicating that our method could detect the lethal species of *A*. sect. *Phalloideae*. Obvious fluorescence signals and amplification curves were absent for the non-lethal subgroup within *A*. sect. *Phalloideae* (No. 6–7), species of the genus *Amanita* not belonging to *A*. sect. *Phalloideae* (No. 8–16), toxic mushroom species belonging to other genera (No. 17–23), and NTC. This showed that our method exhibited strong specificity toward the lethal subgroup of *A*. sect. *Phalloideae*.

### 3.3 Validation of applicability

Real-time PCR was performed with the ten samples listed in Table 3A. As shown in Figure 4B, fluorescence signals and distinct amplification curves were obtained for all samples (no fluorescence signals and an indistinct amplification curve for NTC). This showed that the method exhibited good detection ability when used in samples sequentially subjected to a maximum of 60 min boiling and 120 min simulated digestion with artificial gastric fluid. Therefore, this method possessed good applicability to samples that had been boiled and digested.

### 3.4 Validation of sensitivity

The sensitivity of the developed method to DNA content was evaluated using prepared samples with known DNA concentrations (Table 3B). At an *Amanita pallidorosea* DNA template concentration of 1 pg./μL, the cycle threshold (Ct) values of the amplification curves from the three repeat experiments showed significant variation, were > 30, and strong fluorescence signals were not observed. For *A*. *pallidorosea* DNA template concentrations of 100 ng/μL–10 pg./μL, fluorescence signals, and distinct amplification curves were observed (Figure 4C). Therefore, 10 pg./μL could be regarded as the minimum detection limit for the real-time fluorescence PCR method established in this study.

Real-time PCR performed on serial dilutions of the *Amanita pallidorosea* DNA template also revealed that the Ct value decreased with an increase in DNA template concentration within a certain concentration range. This indicated that DNA template concentration was negatively correlated with the Ct value. Figure 4D shows the constructed standard curve, with the equation of the curve determined as *y* = −2.23992x + 23.82107 (*R*^2^ = 0.9963). It showed a good linear relationship between DNA template concentration and Ct value. The high *R*^2^ also indicates the reliability of the sensitivity of the method.

## 4 Discussion

### 4.1 Section specific nucleotide signature

Previous studies on the nucleotide signatures of macrofungi have generally focused on the screening of signatures of single species. Specific short segments of the species can undoubtedly be obtained through such a screening approach, and species-level nucleotide signatures also exhibit good applicability from the perspective of species-specific identification (Liu et al., 2016; Jiang et al., 2019; Guo et al., 2020). However, macrofungal species belonging to a certain taxonomic group usually exhibit similar traits. The establishment of nucleotide signatures for individual macrofungal species will therefore involve a colossal workload. Sections are often used for the delineation of extremely large genera (Stuessy, 2009). Inspired by the division of the genus *Amanita* into sections, we attempted to seek a nucleotide signature for *Amanita* sect. *Phalloideae* within the genus *Amanita*, which includes highly toxic mushroom species. Ultimately, we obtained a short sequence fragment applicable to all species within the section that could be used as a nucleotide signature. This meant species within *A*. sect. *Phalloideae* could be identified without screening the nucleotide signatures of individual species within the section It is possible to determine whether a species belongs to *A*. sect. *Phalloideae* with a single 17 bp sequence fragment. The development of universal nucleotide signatures at the section level has addressed the limitation of single-species identification associated with traditional molecular identification systems. Specific single nucleotide variations of the lethal *Amanita* subgroup within the section greatly ensure the specificity of subsequent molecular identification. This can assist healthcare units in providing targeted treatment during poisoning incidents caused by *A*. sect. *Phalloideae*, a taxonomic group that consists of lethal mushroom species.

### 4.2 Detection of lethal subgroups within a section

*Amanita* sect. *Phalloideae* is commonly known as the lethal *Amanita* taxon. In many studies centered on this taxon, specific primers have been designed to detect *A. phalloides*, *A. virosa*, and *A. verna* (Gausterer et al., 2014). Researchers have utilized loop-mediated isothermal amplification and hyperbranched rolling circle amplification for the detection of *A*. sect. *Phalloideae* and achieved promising experimental results (He et al., 2019). Phylogenetic and toxin studies related to the section have investigated both the lethal and non-lethal *Amanita* subgroups (Cai et al., 2014). These studies have been targeted toward the detection of certain species or the detection of *A*. sect. *Phalloideae*, but a specifically targeted detection of lethal species in the *Amanita* sect. *Phalloideae* has not yet been reported. Subsequent extensive screening and alignment of the discovered SNPs of this taxon have provided an opportunity to develop a sub-section identification method at the genetic level. This has ultimately enabled species detection within the lethal subgroup of *A*. sect. *Phalloideae*. In addition, the SNP site was found close to the nucleotide signature of *A*. sect. *Phalloideae*. These two findings led to the selection of TaqMan-MGB probe-based real-time PCR to achieve specific detection of the lethal subgroup within *A*. sect. *Phalloideae*. With the high specificity of the nucleotide signature and relatively short length of amplification fragments, this method enabled the detection of lethal species within *A*. sect. *Phalloideae* in samples subjected to extremely harsh conditions (60 min boiling followed by 120 min simulated digestion in artificial gastric fluid). Furthermore, the method did not generate false positive results for the non-lethal subgroups of the section.

Amanitin poisoning clinical symptoms include three major stages: (1) an asymptomatic latency period (6–10 h or rarely 24–36 h); (2) a gastrointestinal phase (lasting 24–48 h); (3) the hepatic-kidney final stage (6–16 days). Many clinical reports describe a “pseudo-remission” before the hepatic-kidney final stage, which should be treated as early as possible, especially gastric lavage, with special attention to “pseudo-remission.” Ultimately, without intense and proper medical care, liver function impairment and kidney damages leading to patient death 6–16 days post-ingestion of amatoxins (Poucheret et al., 2010; Diaz, 2018). The method developed in this study can efficiently and specifically identify the lethal *Amanita* section *Phalloideae* species within 90 min, which can help medical units quickly diagnose and take targeted treatment to save patients in lethal mushroom poisoning.

## 5 Conclusion

In the present study, a nucleotide signature of *Amanita* sect. *Phalloideae* was discovered through extensive screening and an SNP site between the lethal and non-lethal subgroups of *A*. sect. *Phalloideae* was identified. On this basis, we developed a TaqMan-MGB probe-based real-time PCR detection technique for the rapid detection of lethal species within *A*. sect. *Phalloideae*. The detection method demonstrated excellent specificity and was solely targeted toward species belonging to the lethal subgroup of *A*. sect. *Phalloideae*. Results of applicability testing demonstrated that the method could detect lethal species of *A*. sect. *Phalloideae* in samples subjected to simulated gastric digestion of 60 min boiling followed by 120 min incubation in artificial gastric fluid. Subsequent sensitivity testing showed that the detection limit of the method was 10 pg./μL. The results indicate that this technique is applicable to detecting toxic species in real-life poisoning incidents of consumption of toxic mushrooms. Our detection method provides a valuable tool for toxic mushrooms identification in healthcare and disease control units.

## Data availability statement

The datasets presented in this study can be found in online repositories. The names of the repository/repositories and accession number(s) can be found in the article/Supplementary material.

## Author contributions

RD: Visualization, Writing – original draft, Writing – review & editing. JH: Investigation, Project administration, Resources, Supervision, Validation, Writing – review & editing. DZ: Investigation, Supervision, Validation, Writing – review & editing. ET: Data curation, Formal analysis, Funding acquisition, Resources, Supervision, Validation, Writing – review & editing.
