# Supplementary material for: Identification of lethal species in amanita section Phalloideae based on nucleotide signature and specific TaqMan-MGB probe and primer
Source: Front Microbiol. 2024 Feb 1;15:1301085. doi: 10.3389/fmicb.2024.1301085 (PMC10867329; doi:10.3389/fmicb.2024.1301085)
Supplement: Supplementary file 1 [file Table_1.DOC]

| NO. | Species | GenBank | Source |
| --- | --- | --- | --- |
| 1 | *Amanita ballerina* | KY747466.1 | NCBI |
| 2 | *Amanita ballerina* | KY747467.1 | NCBI |
| 3 | *Amanita bisporigera* | OP743456.1 | NCBI |
| 4 | *Amanita bisporigera* | EU819411.1 | NCBI |
| 5 | *Amanita brunneitoxicaria* | KY747463.1 | NCBI |
| 6 | *Amanita brunneitoxicaria* | NR_151655.1 | NCBI |
| 7 | Amanita exitialis | KT213698.1 | NCBI |
| 8 | Amanita exitialis | KT213699.1 | NCBI |
| 9 | Amanita exitialis | KT213700.1 | NCBI |
| 10 | Amanita exitialis | KT213703.1 | NCBI |
| 11 | Amanita exitialis | KT213706.1 | NCBI |
| 12 | Amanita exitialis | KC755037.1 | NCBI |
| 13 | Amanita exitialis | KR996716.1 | NCBI |
| 14 | Amanita exitialis | KR996717.1 | NCBI |
| 15 | Amanita exitialis | KT003192.1 | NCBI |
| 16 | Amanita exitialis | AY855212.1 | NCBI |
| 17 | Amanita exitialis | JX998025.1 | NCBI |
| 18 | Amanita exitialis | JX998026.1 | NCBI |
| 19 | Amanita exitialis | JX998027.1 | NCBI |
| 20 | Amanita exitialis | KT213701.1 | NCBI |
| 21 | Amanita exitialis | KT213702.1 | NCBI |
| 22 | Amanita exitialis | KT213705.1 | NCBI |
| 23 | Amanita exitialis | KT213704.1 | NCBI |
| 24 | Amanita exitialis | KF535949.1 | NCBI |
| 25 | Amanita exitialis | KF535948.1 | NCBI |
| 26 | Amanita exitialis | MN919347.1 | NCBI |
| 27 | Amanita exitialis | MN919346.1 | NCBI |
| 28 | Amanita exitialis | MN919345.1 | NCBI |
| 29 | Amanita exitialis | KJ466375.1 | NCBI |
| 30 | Amanita exitialis | AY436454.1 | NCBI |
| 31 | Amanita franzii | MW032434.1 | NCBI |
| 32 | Amanita fuliginea | FJ176718.1 | NCBI |
| 33 | Amanita fuliginea | FJ176716.1 | NCBI |
| 34 | Amanita fuliginea | JX998022.1 | NCBI |
| 35 | Amanita fuliginea | JX998023.1 | NCBI |
| 36 | Amanita fuliginea | MN061271.1 | NCBI |
| 37 | Amanita fuliginea | KJ466377.1 | NCBI |
| 38 | Amanita fuliginea | KT003189.1 | NCBI |
| 39 | Amanita fuliginea | KT003190.1 | NCBI |
| 40 | Amanita fuliginea | KU356798.1 | NCBI |
| 41 | Amanita fuliginea | KR996718.1 | NCBI |
| 42 | Amanita fuliginea | DQ072730.1 | NCBI |
| 43 | Amanita fuliginea | JX998021.1 | NCBI |
| 44 | Amanita fuliginea | KU601412.1 | NCBI |
| 45 | Amanita fuliginea | KP004944.1 | NCBI |
| 46 | Amanita fuliginea | MW464124.1 | NCBI |
| 47 | Amanita fuliginea | MH508369.1 | NCBI |
| 48 | Amanita fuliginea | KT894847.1 | NCBI |
| 49 | Amanita fuliginea | MW380419.1 | NCBI |
| 50 | Amanita fuliginea | ON115266.1 | NCBI |
| 51 | Amanita fuliginea | ON115267.1 | NCBI |
| 52 | Amanita fuliginea | ON115268.1 | NCBI |
| 53 | Amanita fuliginea | KT213707.1 | NCBI |
| 54 | Amanita fuliginea | KR870315.1 | NCBI |
| 55 | Amanita fuligineoides | ON556403.1 | NCBI |
| 56 | Amanita fuligineoides | ON556404.1 | NCBI |
| 57 | Amanita fuligineoides | FJ176720.1 | NCBI |
| 58 | Amanita fuligineoides | FJ176721.1 | NCBI |
| 59 | Amanita fuligineoides | JX998024.1 | NCBI |
| 60 | Amanita fuligineoides | KC755030.1 | NCBI |
| 61 | Amanita fuligineoides | ON971240.1 | NCBI |
| 62 | Amanita fuligineoides | KP691685.1 | NCBI |
| 63 | Amanita fuligineoides | KP691686.1 | NCBI |
| 64 | Amanita fuligineoides | KY747468.1 | NCBI |
| 65 | Amanita fuligineoides | KY747460.1 | NCBI |
| 66 | Amanita griseorosea | NR_147634.1 | NCBI |
| 67 | *Amanita griseorosea* | KJ466413.1 | NCBI |
| 68 | *Amanita griseorosea* | KJ466411.1 | NCBI |
| 69 | *Amanita griseorosea* | KJ466412.1 | NCBI |
| 70 | *Amanita hesleri* | MT036486.1 | NCBI |
| 71 | *Amanita hesleri* | MH836568.1 | NCBI |
| 72 | Amanita molliuscula | MN061272.1 | NCBI |
| 73 | *Amanita molliuscula* | NR_147633.1 | NCBI |
| 74 | *Amanita ocreata* | KJ466381.1 | NCBI |
| 75 | Amanita pallidorosea | KJ466389.1 | NCBI |
| 76 | Amanita pallidorosea | MH508485.1 | NCBI |
| 77 | Amanita pallidorosea | KT894837.1 | NCBI |
| 78 | Amanita pallidorosea | KU139504.1 | NCBI |
| 79 | Amanita pallidorosea | MN794878.1 | NCBI |
| 80 | Amanita pallidorosea | KU139509.1 | NCBI |
| 81 | Amanita pallidorosea | KU139508.1 | NCBI |
| 82 | Amanita pallidorosea | KU139507.1 | NCBI |
| 83 | Amanita pallidorosea | KU139503.1 | NCBI |
| 84 | Amanita pallidorosea | KU139502.1 | NCBI |
| 85 | Amanita pallidorosea | KF245917.1 | NCBI |
| 86 | Amanita pallidorosea | KT894838.1 | NCBI |
| 87 | Amanita pallidorosea | FJ176734.1 | NCBI |
| 88 | Amanita pallidorosea | MW425335.1 | NCBI |
| 89 | Amanita pallidorosea | ON059315.1 | NCBI |
| 90 | Amanita pallidorosea | FJ176736.1 | NCBI |
| 91 | Amanita pallidorosea | KY616969.1 | NCBI |
| 92 | Amanita pallidorosea | KY616971.1 | NCBI |
| 93 | Amanita pallidorosea | KJ466382.1 | NCBI |
| 94 | Amanita pallidorosea | KY626178.1 | NCBI |
| 95 | Amanita pallidorosea | KJ466386.1 | NCBI |
| 96 | Amanita pallidorosea | KJ466387.1 | NCBI |
| 97 | Amanita pallidorosea | KJ466388.1 | NCBI |
| 98 | Amanita pallidorosea | KJ739814.1 | NCBI |
| 99 | Amanita pallidorosea | KJ739813.1 | NCBI |
| 100 | Amanita pallidorosea | KC755033.1 | NCBI |
| 101 | Amanita pallidorosea | KM052521.1 | NCBI |
| 102 | Amanita pallidorosea | KY621476.1 | NCBI |
| 103 | Amanita pallidorosea | MN061274.1 | NCBI |
| 104 | Amanita pallidorosea | MN794879.1 | NCBI |
| 105 | Amanita pallidorosea | KU139506.1 | NCBI |
| 106 | Amanita pallidorosea | MH508484.1 | NCBI |
| 107 | Amanita pallidorosea | KX270316.1 | NCBI |
| 108 | Amanita pallidorosea | KT779082.1 | NCBI |
| 109 | Amanita pallidorosea | KJ466383.1 | NCBI |
| 110 | Amanita pallidorosea | FJ176735.1 | NCBI |
| 111 | Amanita pallidorosea | JX998035.1 | NCBI |
| 112 | Amanita pallidorosea | JX998036.1 | NCBI |
| 113 | Amanita pallidorosea | JX998037.1 | NCBI |
| 114 | Amanita pallidorosea | KJ466385.1 | NCBI |
| 115 | Amanita pallidorosea | KU139505.1 | NCBI |
| 116 | Amanita pallidorosea | MW425336.1 | NCBI |
| 117 | Amanita pallidorosea | MW554149.1 | NCBI |
| 118 | Amanita pallidorosea | KJ466384.1 | NCBI |
| 119 | Amanita pallidorosea | MW862303.1 | NCBI |
| 120 | Amanita pallidorosea | KF535947.1 | NCBI |
| 121 | Amanita pallidorosea | KY616970.1 | NCBI |
| 122 | Amanita pallidorosea | KY616968.1 | NCBI |
| 123 | Amanita pallidorosea | KF245915.1 | NCBI |
| 124 | Amanita pallidorosea | KU311697.1 | NCBI |
| 125 | Amanita pallidorosea | KU311693.1 | NCBI |
| 126 | *Amanita phalloides* | MZ647957.1 | NCBI |
| 127 | *Amanita phalloides* | KX449201.1 | NCBI |
| 128 | Amanita pseudogemmata | MK239258.1 | NCBI |
| 129 | Amanita rimosa | KF479044.1 | NCBI |
| 130 | Amanita rimosa | MH508547.1 | NCBI |
| 131 | Amanita rimosa | FJ176728.1 | NCBI |
| 132 | Amanita rimosa | MN061275.1 | NCBI |
| 133 | Amanita rimosa | JX998020.1 | NCBI |
| 134 | Amanita rimosa | KF535945.1 | NCBI |
| 135 | Amanita rimosa | KJ466394.1 | NCBI |
| 136 | Amanita rimosa | KU904820.1 | NCBI |
| 137 | Amanita rimosa | KJ466392.1 | NCBI |
| 138 | Amanita rimosa | KJ466393.1 | NCBI |
| 139 | Amanita rimosa | KU904819.1 | NCBI |
| 140 | Amanita rimosa | JX998019.1 | NCBI |
| 141 | Amanita rimosa | KU518317.1 | NCBI |
| 142 | Amanita rimosa | JX998018.1 | NCBI |
| 143 | *Amanita suballiacea* | KJ466420.1 | NCBI |
| 144 | *Amanita suballiacea* | KP221303.1 | NCBI |
| 145 | Amanita subfuliginea | MN061276.1 | NCBI |
| 146 | Amanita subfuliginea | MH142183.1 | NCBI |
| 147 | *Amanita subfuliginea* | NR_147632.1 | NCBI |
| 148 | Amanita subjunquillea | KC755036.1 | NCBI |
| 149 | Amanita subjunquillea | ON059321.1 | NCBI |
| 150 | Amanita subjunquillea | KT894848.1 | NCBI |
| 151 | Amanita subjunquillea | KJ466428.1 | NCBI |
| 152 | Amanita subjunquillea | KJ466427.1 | NCBI |
| 153 | Amanita subjunquillea | MF398993.1 | NCBI |
| 154 | Amanita subjunquillea | MF398992.1 | NCBI |
| 155 | Amanita subjunquillea | MF398991.1 | NCBI |
| 156 | Amanita subjunquillea | KJ466423.1 | NCBI |
| 157 | Amanita subjunquillea | FJ176733.1 | NCBI |
| 158 | Amanita subjunquillea | MW862304.1 | NCBI |
| 159 | Amanita subjunquillea | MZ027338.1 | NCBI |
| 160 | Amanita subjunquillea | KT779084.1 | NCBI |
| 161 | Amanita subjunquillea | MN794889.1 | NCBI |
| 162 | Amanita subjunquillea | JX998034.1 | NCBI |
| 163 | Amanita subjunquillea | MH998628.1 | NCBI |
| 164 | Amanita subjunquillea | MH998629.1 | NCBI |
| 165 | Amanita subjunquillea | MH508622.1 | NCBI |
| 166 | Amanita subjunquillea | MH508624.1 | NCBI |
| 167 | Amanita subjunquillea | KJ466422.1 | NCBI |
| 168 | Amanita subjunquillea | FJ176731.1 | NCBI |
| 169 | Amanita subjunquillea | JX998033.1 | NCBI |
| 170 | Amanita subjunquillea | KJ466424.1 | NCBI |
| 171 | Amanita subjunquillea | KJ466425.1 | NCBI |
| 172 | Amanita subjunquillea | KJ466426.1 | NCBI |
| 173 | Amanita subjunquillea | FJ375332.1 | NCBI |
| 174 | Amanita subjunquillea | DQ072729.1 | NCBI |
| 175 | Amanita subjunquillea | EF442101.1 | NCBI |
| 176 | Amanita subjunquillea | EF442100.1 | NCBI |
| 177 | Amanita subjunquillea | EF442102.1 | NCBI |
| 178 | Amanita subjunquillea | EF442103.1 | NCBI |
| 179 | Amanita subjunquillea | EF442104.1 | NCBI |
| 180 | Amanita subjunquillea | EF442105.1 | NCBI |
| 181 | Amanita subjunquillea | EF442109.1 | NCBI |
| 182 | Amanita subjunquillea | EF442106.1 | NCBI |
| 183 | Amanita subjunquillea | EF442110.1 | NCBI |
| 184 | Amanita subjunquillea | KR996715.1 | NCBI |
| 185 | Amanita subjunquillea | MH998627.1 | NCBI |
| 186 | Amanita subjunquillea | KC414268.1 | NCBI |
| 187 | Amanita subjunquillea | EF442107.1 | NCBI |
| 188 | *Amanita subpallidorosea* | NR_151653.1 | NCBI |
| 189 | *Amanita subpallidorosea* | KP691683.1 | NCBI |
| 190 | Amanita virosa | KT697955.1 | NCBI |
| 191 | Amanita virosa | KT697946.1 | NCBI |
| 192 | Amanita virosa | KT697942.1 | NCBI |
| 193 | Amanita virosa | KT697945.1 | NCBI |
| 194 | Amanita virosa | KT697948.1 | NCBI |
| 195 | Amanita virosa | KT697939.1 | NCBI |
| 196 | Amanita virosa | OM451556.1 | NCBI |
| 197 | Amanita virosa | MG516218.1 | NCBI |
| 198 | Amanita virosa | MZ647955.1 | NCBI |
| 199 | Amanita virosa | KT894836.1 | NCBI |
| 200 | Amanita virosa | KJ466430.1 | NCBI |
| 201 | Amanita virosa | JX998030.1 | NCBI |
| 202 | Amanita virosa | JX998029.1 | NCBI |
| 203 | Amanita virosa | JX998028.1 | NCBI |
| 204 | Amanita virosa | FJ755188.1 | NCBI |
| 205 | Amanita virosa | FJ176737.1 | NCBI |
| 206 | Amanita virosa | AB015676.1 | NCBI |
| 207 | Amanita virosa | GU373492.1 | NCBI |
| 208 | Amanita virosa | KY924845.1 | NCBI |
| 209 | Amanita virosa | KR862367.1 | NCBI |
| 210 | Amanita virosa | KJ466429.1 | NCBI |
| 211 | Amanita virosa | MH508650.1 | NCBI |
| 212 | Amanita virosa | KJ466431.1 | NCBI |
| 213 | Amanita virosa | KY472227.1 | NCBI |
| 214 | Amanita virosa | MT345282.1 | NCBI |
| 215 | Amanita virosa | KM373251.1 | NCBI |
| 216 | Amanita virosa | EU909449.1 | NCBI |
| 217 | Amanita virosa | KJ638290.1 | NCBI |
| 218 | Amanita virosa | KJ638289.1 | NCBI |
| 219 | Amanita virosa | KF937304.1 | NCBI |
| 220 | Amanita virosa | KT697986.1 | NCBI |
| 221 | Amanita virosa | EU909450.1 | NCBI |
| 222 | Amanita zangii | MH508655.1 | NCBI |
| 223 | Amanita zangii | KU904817.1 | NCBI |
| 224 | Amanita zangii | KU904818.1 | NCBI |
| 225 | Amanita zangii | KY747470.1 | NCBI |
| 226 | Amanita zangii | KY747472.1 | NCBI |
| 227 | Amanita zangii | KJ466432.1 | NCBI |
| 228 | Amanita zangii | KJ466433.1 | NCBI |
| 229 | *Pluteus rangifer* | KJ009650.1 | NCBI |
| 230 | *Pluteus sepiicolor* | KJ009765.1 | NCBI |
| 231 | Amanita pseudogemmata | OQ983890 | Self-test |
| 232 | Amanita molliuscula | OQ983891 | Self-test |
| 233 | Amanita molliuscula | OQ983892 | Self-test |
| 234 | Amanita fuliginea | OQ983893 | Self-test |
| 235 | Amanita fuliginea | OQ983894 | Self-test |
| 236 | Amanita subjunquillea | OQ983895 | Self-test |
| 237 | Amanita exitialis | OQ983896 | Self-test |
| 238 | Amanita pallidorosea | OQ983897 | Self-test |
| 239 | Amanita pallidorosea | OQ983898 | Self-test |
| 240 | Amanita pallidorosea | OQ983899 | Self-test |
| 241 | Amanita pallidorosea | OQ983900 | Self-test |
| 242 | Amanita pallidorosea | OQ983901 | Self-test |
| 243 | Amanita pallidorosea | OQ983902 | Self-test |
| 244 | Amanita molliuscula | OR121068 | Self-test |
| 245 | Amanita molliuscula | OR121069 | Self-test |
| 246 | Amanita molliuscula | OR121070 | Self-test |
| 247 | Amanita molliuscula | OR121071 | Self-test |
| 248 | Amanita molliuscula | OR121072 | Self-test |
| 249 | Amanita molliuscula | OR121073 | Self-test |
| 250 | Amanita molliuscula | OR121074 | Self-test |
| 251 | Amanita molliuscula | OR121075 | Self-test |
| 252 | Amanita molliuscula | OR121076 | Self-test |
| 253 | Amanita molliuscula | OR121077 | Self-test |
| 254 | Amanita pallidorosea | OR121078 | Self-test |
| 255 | Amanita pallidorosea | OR121079 | Self-test |
| 256 | Amanita pallidorosea | OR121080 | Self-test |
| 257 | Amanita pallidorosea | OR121081 | Self-test |
| 258 | Amanita pallidorosea | OR121082 | Self-test |
| 259 | Amanita subjunquillea | OR121083 | Self-test |
| 260 | Amanita subjunquillea | OR121084 | Self-test |
| 261 | Amanita subjunquillea | OR121085 | Self-test |
| 262 | Amanita subjunquillea | OR121086 | Self-test |
| 263 | Amanita subjunquillea | OR121087 | Self-test |
| 264 | Amanita subjunquillea | OR121088 | Self-test |
| 265 | Amanita subjunquillea | OR121089 | Self-test |
| 266 | Amanita subjunquillea | OR121090 | Self-test |
| 267 | Amanita subjunquillea | OR121091 | Self-test |
| 268 | Amanita subjunquillea | OR121092 | Self-test |

**Supplementary Table S1.** Sequences used for sequence alignment.
